# Neuroanatomical correlates of poststroke complex regional pain syndrome: a voxel-based lesion symptom-mapping study

**DOI:** 10.1038/s41598-021-92564-7

**Published:** 2021-06-22

**Authors:** Jae-Ik Lee, Soon-Woo Kwon, Ahry Lee, Woo-suk Tae, Sung-Bom Pyun

**Affiliations:** 1 Department of Physical Medicine and Rehabilitation, Anam Hospital, Korea University College of Medicine, Seoul, Republic of Korea; 2 Brain Convergence Research Center, Korea University College of Medicine, Seoul, Republic of Korea; 3 Department of Biomedical Sciences, Korea University College of Medicine, Seoul, Republic of Korea

**Keywords:** Anatomy, Neurology, Signs and symptoms

## Abstract

Complex regional pain syndrome (CRPS) is a common poststroke complication. However, the neural substrates associated with CRPS remain unclear. We investigated the neural correlates associated with poststroke CRPS using voxel-based lesion‒symptom mapping (VLSM) analysis. Among 145 patients with ischemic stroke, 35 were diagnosed with CRPS and categorized into the poststroke CRPS group, and the remaining 110 into the control group. We compared the clinical characteristics between the groups. VLSM analysis was performed to identify the brain region associated with the development of poststroke CRPS. The clinical findings suggested that the poststroke CRPS group had lower muscle strength; lower scores on Fugl‒Meyer assessment, Manual Function Test, Mini-Mental Status Examination; and higher incidence of absent somatosensory evoked potentials in the median nerve than the control group. The head of the caudate nucleus, putamen, and white matter complexes in the corona radiata were significantly associated with poststroke CRPS development in ischemic stroke patients. These results facilitate an understanding of poststroke CRPS pathophysiology. Monitoring patients with lesions in these structures may aid the prevention and early treatment of poststroke CRPS.

Complex regional pain syndrome (CRPS) is a clinical syndrome that typically manifests with severe pain in one or more extremities, with vasomotor, sensory, motor, and trophic changes^1,2^. Although the pathogenesis of CRPS is unclear, most poststroke CRPS is considered to be type I, previously known as reflex sympathetic dystrophy. CRPS type I involves severe pain and disability without obvious evidence of peripheral nerve injury in the affected extremity. Previous studies have reported that the incidence of CRPS among patients with poststroke hemiplegia was as high as 50% during a 28-week follow-up period^3^. Poststroke CRPS usually occurs 1‒6 months after stroke diagnosis, which is the period with the highest potential for rehabilitation^4^. Therefore, early diagnosis and treatment of poststroke CRPS are essential for successful rehabilitation after stroke.

Previous studies identified subluxation of the shoulder, immobilization of the upper extremity, severity of motor deficits and joint trauma, and rotator cuff tear, among several other, as the factors predisposing patients to poststroke CRPS^5–7^. However, the pathophysiology and structural lesions in the brain responsible for poststroke CRPS are not well understood. In a previous study^8^, the grey matter volume in the posterior midcingulate cortex, bilateral pregenual anterior cingulate cortex, orbitofrontal cortex, and left posterior insula was smaller in the a poststroke CRPS group than in the healthy control group on lesion analysis using structural magnetic resonance images. An analysis using several neuroimaging studies reported that basal ganglia (BG) dysfunction plays an important role in the development of CRPS^9^. However, only a few studies have directly investigated the neuroanatomical correlates associated with poststroke CRPS. Voxel-based lesion‒symptom mapping (VLSM) is a lesion‒symptom analysis technique that provides information about the association between the locations of brain lesions and patient behavior^10^. To date, only one study has used VLSM analysis to identify the lesion responsible for the development of poststroke CRPS^11^. The study revealed that poststroke CRPS could be associated with damage to the corticospinal tract (CST) and the adjacent lentiform nucleus. However, that study had some limitations. An age-matched control group was used for VLSM analysis, which could pose a risk of selection bias. Patients with both ischemic and haemorrhagic stroke were included in the study, and there were more patients with haemorrhagic stroke. Accurate delineation of the haemorrhagic stroke lesion using VLSM analysis can be affected over time after the onset of stroke, and intracerebral haemorrhages usually occur in the BG and thalamic structures^12^.

Therefore, the primary objective of this study was to determine the neural correlates of poststroke CRPS using VLSM analysis in all patients with ischemic stroke, with or without poststroke CRPS, during the same period of recruitment.

## Materials and methods

### Subjects

We retrospectively enrolled stroke patients admitted to the rehabilitation department of Korea University Anam Hospital from November 2012 to August 2019. The diagnosis of ischemic stroke was based on clinical history and neurological examination findings, and was confirmed by brain magnetic resonance imaging (MRI) or computed tomography. The inclusion criteria for stroke patients were as follows: (1) first-ever episode of ischemic stroke and (2) unilateral hemispheric, supratentorial lesion. The exclusion criteria were as follows: (1) intracerebral hemorrhage, (2) bilateral hemispheric or infratentorial lesions, (3) history of previous brain disorders, such as traumatic brain injury, brain tumour, and other degenerative brain disorders, (4) history of peripheral neuropathy, neuromuscular disorders, or musculoskeletal deformity in the upper extremities, and (5) history of pain or ongoing chronic pain.

Demographic data of the subjects and the clinical characteristics of their affected arm were obtained. The time duration from the onset of stroke to the MRI scan was collected in all study subjects. Time from stroke onset to diagnosis of CRPS was also collected in the poststroke CRPS group. The Medical Research Council (MRC) scale was used to assess motor strength of shoulder flexion and wrist extension, glenohumeral subluxation, and upper limb spasticity, and the presence of somatosensory evoked potential in the median nerve was also recorded for analysis. The Fugl‒Meyer assessment score was used to evaluate motor function, such as synergy, strength, and coordination, after stroke^13,14^, and the Manual Function Test was used to assess the function of the hemiplegic upper limb after the stroke^15^. In addition, the Berg Balance Scale was used to measure general balance^16^. The Modified Barthel Index was used to assess functional independence^17^. The Korean version of the Mini-Mental State Examination (MMSE) was used to record cognitive function^18^. Furthermore, we investigated the location of the lesion (middle cerebral arterial territory *vs.* other) and measured the lesion volume using brain MRI. The data were compared between the poststroke CRPS and control groups.

This study was approved by the Institutional Review Board (IRB) of the Korea University Anam Hospital (IRB No. 2019AN0322) and was conducted in accordance with the principles of the Declaration of Helsinki. The IRB waived the need to obtain informed consent because this study included a retrospective review of medical records and radiographic images.

### Poststroke CRPS and control group allocation

**Figure 1:**
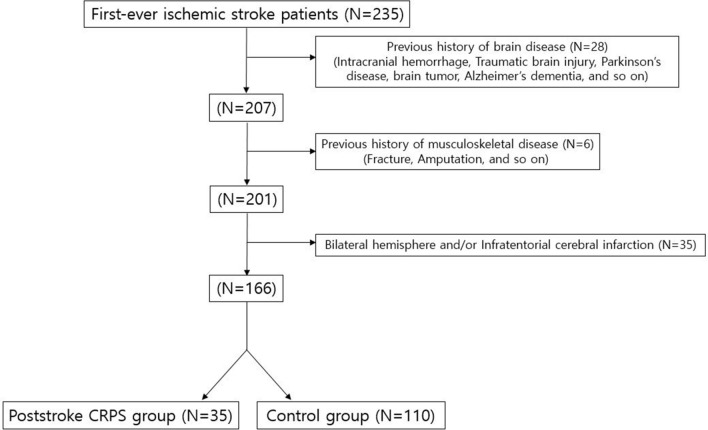
Flow diagram of enrolment of the subjects in the poststroke complex regional pain syndrome (CRPS) and control groups. N means numbers of included subjects. After the classification process, a total of 145 patients were finally included in the study, of which 35 patients and 110 patients were assigned to the poststroke CRPS group and the control group, respectively.

Poststroke CRPS was diagnosed as per the Budapest Criteria of the International Association for the Study of Pain (revised in 2003) that grouped the signs and symptoms into two clusters: sensory and sudomotor/vasomotor changes^19,20^. These patients had continuous pain—disproportionate to any inciting event, had at least one symptom belonging to three of the four categories (e.g., sensory, vasomotor, sudomotor/oedema, motor/trophic), and at least one sign that could be classified in two or more or the abovementioned categories. Moreover, no other diagnosis could better explain the patients’ signs and symptoms^19,20^. Although triple-phase bone scans can help diagnose CRPS, this is not included in the diagnostic criteria. Furthermore, negative triple-phase bone scans do not always rule out CRPS, and their reported sensitivity and specificity vary considerably^21^. Therefore, we did not consider triple-phase bone scan results in the inclusion/exclusion criteria. For statistical purposes, we considered the diagnosis of poststroke CRPS as a binary variable: absent or present. All other patients not included in the poststroke CRPS group were allocated to the control group. After the classification process, 145 patients were finally included in the study; among them, 35 and 110 patients were assigned to the poststroke CRPS group and control group, respectively (Fig. 1).

### MR data acquisition

Patients’ MRI scans were obtained parallel to the anterior commissure–posterior commissure line using 3.0-T MRI scanners. Of the 145 patients, 57 were scanned using an Achieva scanner (Philips Healthcare, Best, the Netherlands), 35 using a TrioTim scanner (Magnetom Trio, Tim System, Siemens), 36 using a Skyra scanner (Siemens), and 17 using a Prisma MRI scanner (Siemens, Erlangen, Germany). All MRI scans were obtained using T1-weighted magnetization-prepared rapid gradient-echo and fluid-attenuated inversion recovery (FLAIR) sequences. T1-weighted (T1W) scans were obtained with the following parameters: repetition time, 1900 ms; echo time, 2.6 ms; field-of-view, 220 mm; matrix size, 256 × 256; slice thickness, 1 mm; 176 coronal slices without a gap; voxels, 0.86 × 0.86 × 1 mm^3^; flip angle, 16°; and number of excitations, 1. The FLAIR scans were obtained with following parameters: repetition time, 9000 ms; field-of-view, 220 mm; matrix size, 384 × 278; slice thickness, 4 mm; and 31 coronal slices without a gap.

### VLSM processing

**Figure 2:**
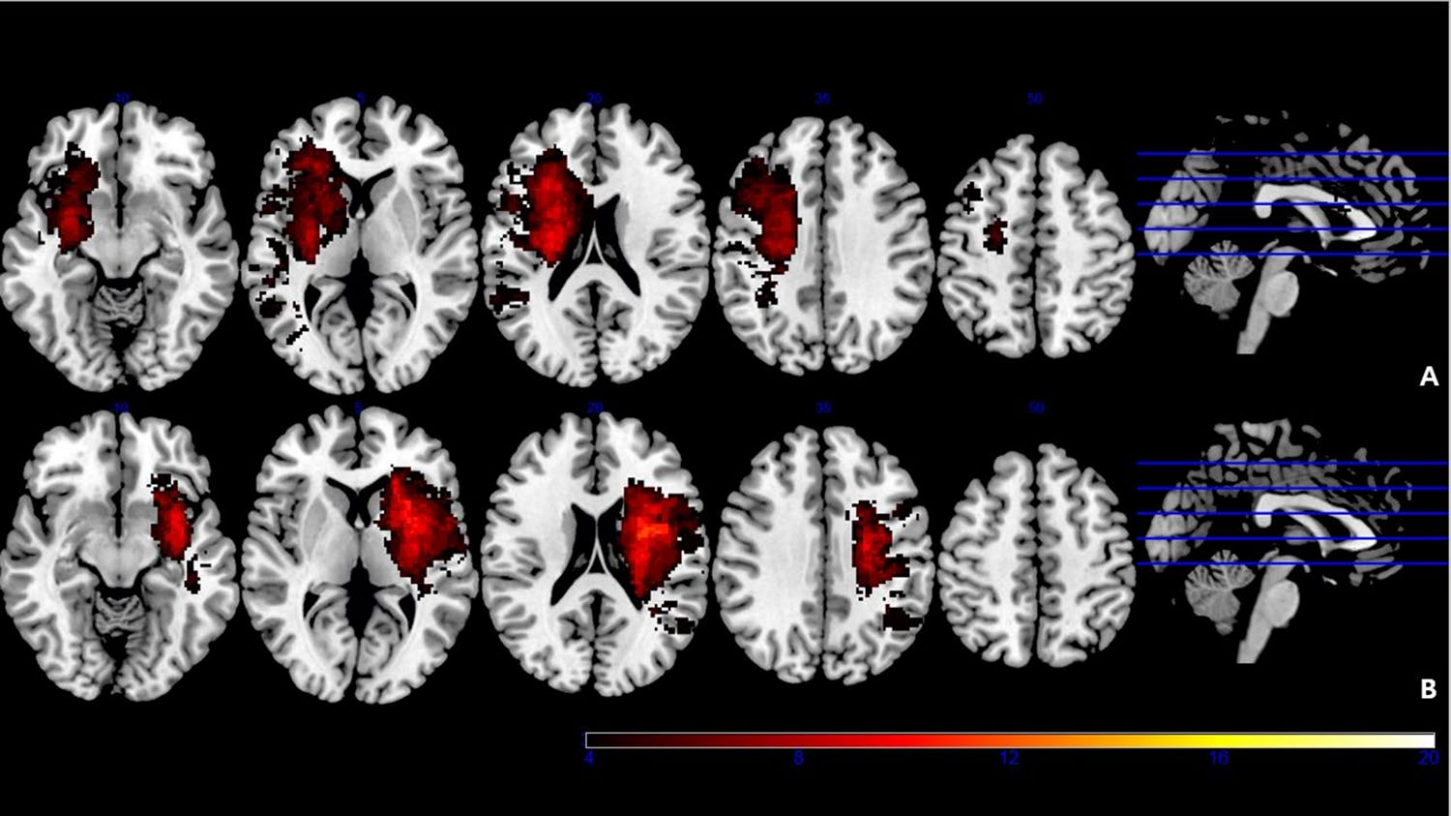
Lesion overlap plot of the poststroke CRPS group, with lesions in the right hemisphere (N = 18) (**A**) and left hemisphere (N = 17) (**B**). The colour bar represents the number of patients with lesions in that area.

Delineation of the lesions was performed by a single physician (J. Lee) for all axially sectioned images. All lesions were displayed using MRIcron software (http://www.mccauslandcenter.sc.edu/mricro/mricron/). Previous studies have presented some evidence to indicate that the non-dominant hemisphere has a lower threshold for pain^22^, and that in children, CRPS shows more frequent left-side lateralization of the lower extremity^23^. However, there are few studies about the hemispheric dominance of poststroke CRPS, and all lesion maps were flipped to the left side to focus our analysis on the localization of the lesions, irrespective of lateralization. Before flipping, we checked the lesion-overlapping plot of poststroke CRPS patients with lesions in the right and left hemispheres and found that there was no significant difference in the distribution of lesions between the right and left hemispheres in the poststroke CRPS group (Fig. 2). After flipping, the lesions were drawn manually on the patients’ FLAIR images. These images were co-registered with the patients’ own T1W images. They were then normalized to Montreal Neurologic Institute (MNI) space using T1W normalization parameters and Statistical Parametric Mapping (SPM) 12 software (Wellcome Department of Neuroscience, London, UK; http://www.fil.ion.ucl.ac.uk/spm/software/spm12/). After normalization, the voxel size was 2 × 2 × 2 mm^3^. Normalized lesions were subjected to statistical mapping analysis using VLSM algorithms implemented with the Statistical Non-Parametric Mapping (SnPM) toolbox (https://warwick.ac.uk/fac/sci/statistics/staff/academic-research/nichols/software/snpm). After considering clinical parameters (age and sex), VLSM analysis revealed clusters of voxels that were significantly and more frequently identified in the poststroke CRPS group than in the control group. Statistical significance was determined by voxel-level permutation correction at *p* < 0.05 family-wise error (FWE) correction with explicit masking of the left hemisphere. The significantly damaged lesions’ MNI coordinates were presented in the SnPM12 program and converted to anatomical regions using xjview software (https://www.alivelearn.net/xjview/). Mean lesion volumes in each group were calculated in three dimensions using the ITK-SNAP program (ver. 3.8.0; http://www.itksnap.org/pmwiki/pmwiki.php).

The spatial relationship between the lesions and the CST was evaluated with the two normalized mask images (lesion and CST) in MNI space overlaid on the MNI152 brain template of MRIcroGL (https://www.mccauslandcenter.sc.edu/mricrogl). Normalized CST images were downloaded from the NatBrainLab (https://www.natbrainlab.co.uk/atlas-maps)^24^.

### Statistical analysis

For statistical analysis of demographic data and clinical characteristics, we used IBM SPSS Statistics, Version 24.0, which was used to derive the mean and standard deviation of both groups. The differences in clinical variables between the two groups were analysed using the Mann‒Whitney U test because none of the clinical variables followed a normal distribution. The chi-square test was used to compare differences in sex, the presence of somatosensory evoked potentials, spasticity, shoulder subluxation, and location of the lesion (middle cerebral arterial territory only *vs.* other) in both groups. The difference in location of the lesion between the two groups was tested for statistical significance using the SnPM program, as mentioned earlier. Statistical significance was set at *p* < 0.05.

## Results

### General characteristics of the subjects

Demographic characteristics and clinical variables of the poststroke CRPS and control groups are shown in Table 1. A standard MRI scan was obtained at 3.00 (2.00–6.00) days after stroke onset in the poststroke CRPS group, and at 3.00 (2.00–5.00) days after stroke onset in the control group (*p* > 0.05). In the poststroke CRPS group, the average time between onset and diagnosis of CRPS was 47.00 (27.00–76.00) days. Scores of the Manual Function Test, Modified Barthel Index, Fugl‒Meyer assessment (total and upper extremity), MRC scale for the shoulder flexion and wrist extension of the hemiplegic side, Berg Balance Scale, and MMSE were significantly lower in the poststroke CRPS than in the control group (*p* < 0.05). Absence of somatosensory evoked potentials in the affected upper extremities was more frequent in the poststroke CRPS than in the control group (*p* = 0.002). Age and sex, presence of shoulder subluxation and spasticity, Modified Barthel Index score, stroke lesion location, and lesion volume showed no significant differences between the two groups.

**Table 1:** General characteristics of subjects.

	CRPS group (N = 35)	Control group (N = 110)	*p*-value
Age (year)	72.00 (65.00–78.00)	72.00 (58.75–80.00)	0.708
Sex (male/female)	17/18	67/43	0.198
K-MMSE (30)	11.00 (0.00–23.00)	21.00 (9.00–27.00)	
Shoulder subluxation (present/absent)	8/27	16/94	0.249
**Muscle strength (MRC grade)**			
Shoulder flexion	1.00 (0.00–2.00)	3.00 (1.00–4.00)	< 0.001*
Wrist extension	1.00 (0.00–2.00)	3.00 (0.00–4.00)	0.004*
**Fugl‒Meyer assessment**			
Upper extremity (66)	4.00 (4.00–6.00)	27.00 (4.00–58.50)	< 0.001*
Total (100)	10.00 (8.00–35.00)	50.00 (10.00 -86.00)	< 0.001*
Manual Function Test (32)	0.00 (0.00–1.00)	11.50 (0.00–24.25)	< 0.001*
Spasticity (present/absent)	6/29	9/101	0.129
SSEP, median nerve (present/absent) †	14/13	84/18	0.002*
Berg Balance Scale (56)	3.00 (0.00–8.00)	9.50 (3.75–39.25)	0.002*
Modified Barthel Index (100)	13.00 (2.00–33.00)	41.50 (13.00–67.00)	0.001*
Lesion location (MCA territory only/Other)	29/6	94/16	0.446
Lesion volume (cc)	60,180.00 (12,350.00–170,400.00)	27,890.00 (4048.00–129,675.00)	0.053
Time from stroke onset to MRI scan (days)	3.00 (2.00–6.00)	3.00 (2.00–5.00)	0.872
Time from stroke onset to diagnosis of CRPS (days)	47.00 (27.00–76.00)	–	–

All ordinary and continuous data were presented as median (interquartile range).

Numbers in parenthesis are maximal score.

MRC, Medical Research Council scale; SSEP, Somatosensory Evoked Potentials; K-MMSE, Korean version of the Mini Mental Status Exam; MCA, Middle Cerebral Artery.

^†^Data were missing for 16 subjects.

### Lesion location associated with poststroke CRPS using VLSM

**Figure 3:**
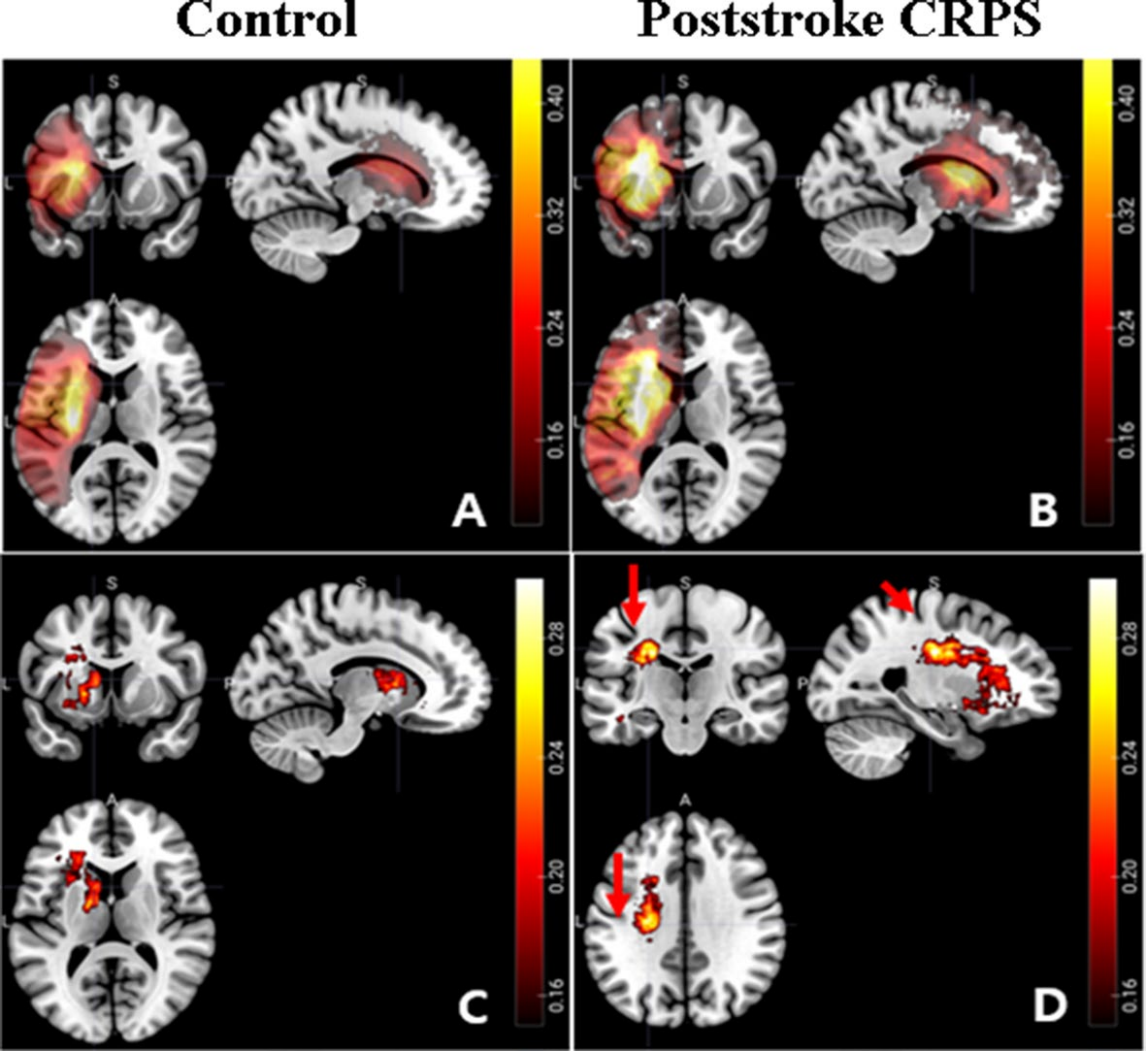
Lesion-overlaid maps of controls and CRPS patients. The colour bar represents the percentage of overlapping lesions (range: 0 [no overlap] to 1 [complete overlap]) (**A**: control group, **B**: poststroke CRPS group) or the subtracted percentage of overlapped lesions (the difference in the percentage of overlapped lesions) from patients in the control and poststroke CRPS groups (**C**: control group, **D**: poststroke CRPS group). The red arrows indicate the central sulcus (**D**). MR images are displayed according to neurological convention. Consequently the head of the caudate nucleus, putamen, and white matter complexes in the corona radiata were more frequently involved in the poststroke CRPS group than in the control group.

**Figure 4:**
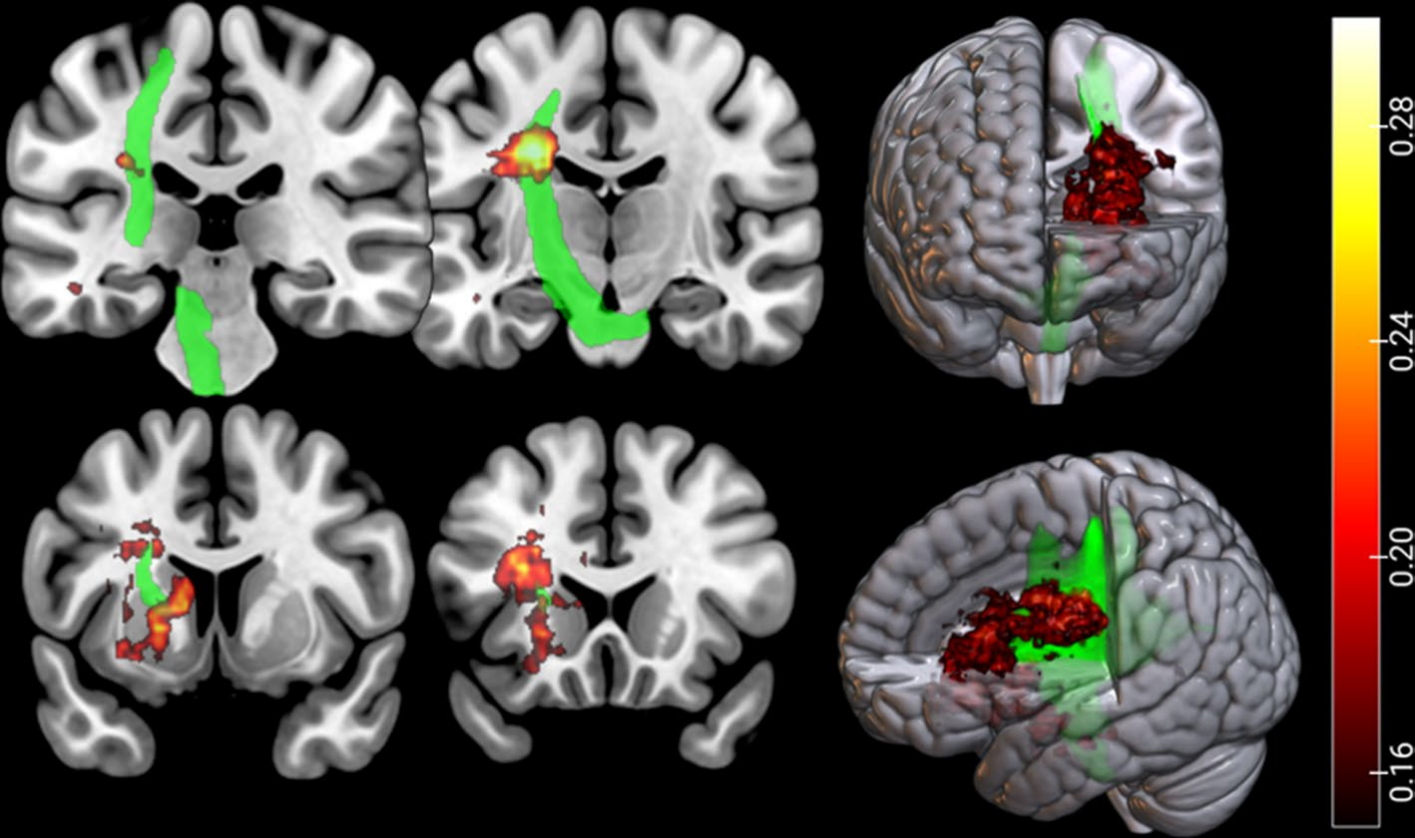
Spatial relationship between the lesion and corticospinal tract. The subtracted percentage map (the difference map of percentage of overlapped lesions) was overlaid on the corticospinal tract (green). The colour bar represents the subtracted percentage of overlapped lesions from the patients in the CRPS and control groups. MR images are displayed according to neurological convention. The lesion site from our experimental results substantially overlapped with the descending pathway of the corticospinal tract.

**Figure 5:**
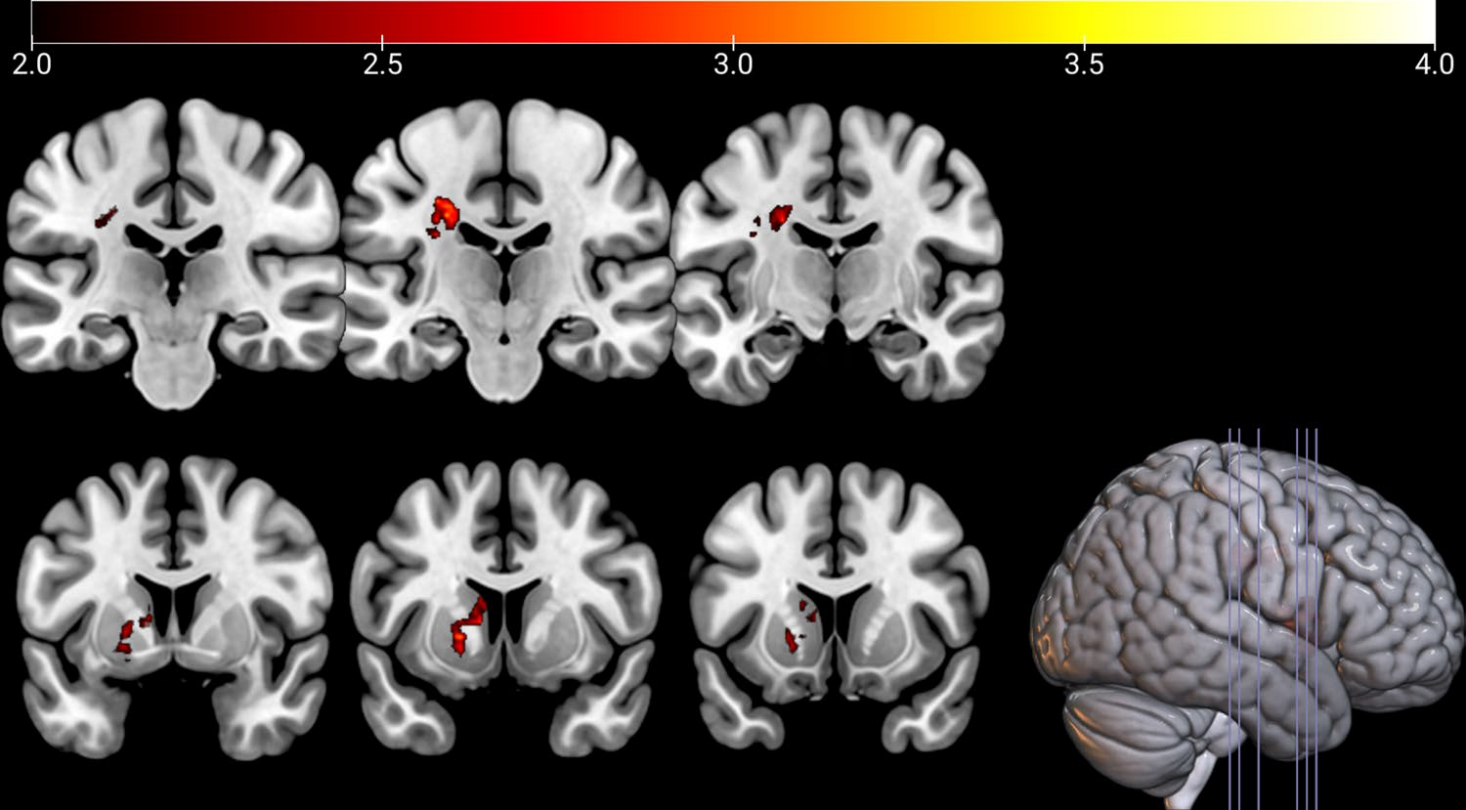
Non-parametric voxel-wise comparison maps. Two non-parametric samples with adjustment for the confounders of age and sex. The colour bar represents the p-value of nonparametric tests for voxel-wise comparison maps from the patients in the CRPS and control groups. CRPS patients showed significant damage in the corticospinal tract of the white matter (local maxima, uncorrected *p* = 0.0002), caudate nucleus, and putamen (local maxima, uncorrected *p* = 0.0006). The colour bar represents *t* values.

The lesion overlay maps for each group are presented in Fig. 3A,B. The voxel-wise comparison maps by subtraction for two samples are presented in Fig. 3C,D. When compared to the previously documented CST pathway (indicated by the green line)^25^, these maps overlap with the CST that governs the motor function (Fig. 4). Furthermore, Fig. 5 presents the voxel-wise comparison maps created by analysis of nonparametric tests for two samples, with adjustment for age and sex confounders. Similar to the simple subtraction map described in Fig. 3C,D, damage to the white matter of the CST (local maxima FWE corrected *p* = 0.03), caudate nucleus, and putamen (local maxima FWE corrected *p* = 0.01) was significantly more frequent in the poststroke CRPS group than in the control group (Fig. 5).

## Discussion

This study was conducted to determine the location of brain lesions that could affect the occurrence of poststroke CRPS in patients with ischemic stroke. Our results suggested that lesions involving the CST and BG, including the putamen and caudate nucleus, are significant neural substrates for the development of poststroke CRPS. The motor strength of the hemiplegic arm, Fugl‒Meyer assessment score (total and upper extremity), Manual Function Test score, Berg Balance score, presence of somatosensory evoked potentials, and MMSE score were significantly lower in the poststroke CRPS group than in the control group.

The strength of this study is the inclusion of a homogenous group of patients with ischemic stroke and a control group with a large sample size, with no selection bias, reinforcing the statistical power of VLSM. We were able to identify that lesions in the head of the caudate nucleus, putamen, and the CST in the corona radiata were significantly associated with poststroke CRPS. These results were similar to those of a recent VLSM study, which reported that the CST and lentiform nucleus were associated with poststroke CRPS^11^. The caudate nucleus and putamen are also known for their role in the pain pathway^9,26^. A previous study using a human functional MRI revealed that, when noxious stimuli were applied, the caudate nucleus was activated bilaterally^27,28^. Although the exact mechanism is unknown, it seems that the caudate nucleus plays an important role in both sensory processing and pain suppression^29^. Therefore, based on the results of this study, it can be concluded that stroke involving the caudate nucleus can inhibit the pain modulation process and contribute to the development of poststroke CRPS.

The white matter complexes in the corona radiata, particularly the CST, serve as a transmission path for motor signals. Motor fibres from the motor cortex travel via the centrum semiovale and corona radiata to converge in the posterior limb of the internal capsule^30^. The positive relationship between the development of poststroke CRPS and the severity of motor impairment of the hemiplegic arm has already been proven in several studies^3,5,6^. In our study, measures related to CST motor function, such as the strength of shoulder flexion and wrist extension, Fugl‒Meyer assessment, and Manual Function Test scores in the affected limb were significantly decreased in the poststroke CRPS group. These clinical findings indicate that, although the pathomechanism is unclear, there is some association between motor weakness due to injury of the corona radiata and the incidence of poststroke CRPS. Further studies are warranted to clarify this relationship.

The somatosensory aspect of the CRPS cannot be ignored. Our study also assessed the white matter fibres projecting to the primary somatosensory cortex and the motor pathway. The dorsal column medial lemniscus pathway, which transmits the sensation of fine touch, two-point discrimination, conscious proprioception, and vibration sensations from the body, travels through the ventral posterolateral nucleus of the thalamus^31^. It reaches the primary somatosensory cortex of the postcentral gyrus via the posterior limb of the internal capsule and projects white matter to the cortex^27^. Several studies have reported that injury to the somatosensory cortex was associated with an increase in sustained nociceptive inputs to the hemiplegic limb suffering from poststroke CRPS^32,33^. Another study revealed that the absence of the median somatosensory evoked potentials in the hemiplegic arm could be a predictor of the onset of poststroke CRPS^34^. Consistent with the results of previous studies^34^, we found that absence of SEP responses in the affected limb was significantly associated with the poststroke CRPS group in this study.

Our study had several limitations. First, although we retrospectively recruited stroke patients over a long period, and the sample size of the poststroke CRPS group was small. Second, although none of the previous studies have clearly elucidated the laterality of brain lesions in CRPS, some studies have shown that chronic pain might be more prevalent on the non-dominant side than on the dominant side^22^. Therefore, flipping of images to a single side in this study may represent a weakness. Thus, future research should delineate whether poststroke CRPS has predominance in one hemisphere. Third, poststroke CRPS was analysed as a binary variable: present or absent. A more meaningful result may have been derived if the analysis had been performed based on the degree of pain or the number of symptoms or signs. However, since this study was conducted retrospectively and because the number of signs and symptoms were not clearly recorded for some patients, we had to analyse this as a binary variable. This caveat will be addressed in future studies. Finally, as an inherent limitation of VLSM analysis, there may have been false-positive lesions due to the similarity between the true lesion and the regions supplied by the same blood vessels^35^.

## Conclusions

Based on our results, we conclude that the head of the caudate nucleus, putamen, and white matter complexes in the corona radiata, particularly the CST, are associated with the development of poststroke CRPS. Our study results may promote understanding of the pathophysiology of poststroke CRPS. Moreover, monitoring stroke patients with lesions in these brain structures may facilitate prevention and early treatment of poststroke CRPS.

## Data Availability

The datasets generated during and/or analysed during the current study are available from the corresponding author on reasonable request.
